# Towards *Miscanthus* combustion quality improvement: the role of flowering and senescence

**DOI:** 10.1111/gcbb.12391

**Published:** 2016-09-30

**Authors:** Elaine Jensen, Paul Robson, Kerrie Farrar, Sian Thomas Jones, John Clifton‐Brown, Roger Payne, Iain Donnison

**Affiliations:** ^1^ Institute of Biological, Environmental and Rural SciencesAberystwyth UniversityPlas GogerddanAberystwyth, CeredigionSY23 3EBWalesUK; ^2^ VSN International Ltd2 Amberside House, Wood LaneHemel HempsteadHP2 4TPUK

**Keywords:** bioenergy, biomass combustion, chemical composition, nutrient remobilization, sustainability

## Abstract

In commercially grown *Miscanthus *× *giganteus*, despite imposing a yield penalty, postwinter harvests improve quality criteria for thermal conversion and crop sustainability through remobilization of nutrients to the underground rhizome. We examined 16 *Miscanthus* genotypes with different flowering and senescence times for variation in N, P, K, moisture, ash, Cl and Si contents, hypothesizing that early flowering and senescence could result in improved biomass quality and/or enable an earlier harvest of biomass (in autumn at peak yield). Ideal crop characteristics at harvest are low N and P to reduce future fertilizer inputs, low K and Cl to reduce corrosion in boilers, low moisture to reduce spoilage and transportation costs, and low Si and ash to reduce slagging and consequent operational downtime. Stems and leaves were harvested during summer, autumn and then the following spring after overwinter ripening. In spring, stem contents of N were 30–60 mg kg^−1^, P were 203–1132 mg kg^−1^, K were 290–4098 mg kg^−1^, Cl were 10–23 mg kg^−1^ and moisture were 12–38%. Notably, late senescence resulted in increased N, P, K, Cl, moisture and ash contents, and should therefore be avoided for thermochemical conversion. Flowering and senescence led to overall improved combustion quality, where flowered genotypes tended towards lower P, K, Cl and moisture contents; marginally less, or similar, N, Si and ash contents; and a similar higher heating value, compared to those that had not flowered. Such genotypes could potentially be harvested in the autumn. However, one genotype that did not flower in our trial exhibited sufficiently low N and K content in autumn to meet the EN *plus* wood pellet standards for those traits, and some of the lowest P, moisture and ash contents in our trial, and is thus a target for future research and breeding.

## Introduction

*Miscanthus* is a perennial energy crop with C4 metabolism. It can produce high yields from low inputs across multiple environments in temperate regions as well as in the tropics (Mccalmont *et al*., 2015). The most cultivated *Miscanthus* species for biomass production in Europe and North America is *M*. × *giganteus*, which exhibits rapid growth, low mineral content and high yield (Linde‐Laursen, 1993; Lewandowski & Kicherer, 1997). *Miscanthus* × *giganteus* resulted from a cross in the wild between diploid *M. sinensis* (2*n* = 2*x* = 38) and tetraploid *M. sacchariflorus* (2*n* = 4*x* = 76) (Greef & Deuter, 1993; Linde‐Laursen, 1993; Rayburn *et al*., 2009) and is sterile, prohibiting improvement through breeding. However, the *Miscanthus* genus comprises 13 or so species (Greef & Deuter, 1993; Hodkinson *et al*., 1997) of high diversity, providing considerable genetic and phenotypic resources to improve *Miscanthus* both in terms of quality and quantity of harvested biomass. Currently the crop is grown for heat and power, although in the wider bioeconomy there is also interest in its use for green chemistry (Parveen *et al*., 2011), biomaterials (Uihlein *et al*., 2008) and transport fuels (Brosse *et al*., 2012).

During combustion, the physical and chemical properties of the biomass itself, such as lignin, cellulose, hemicellulose, moisture content and elemental composition, strongly influence factors such as calorific value, ash and its behaviour, heat exchange and emissions (Robbins *et al*., 2012; Shao *et al*., 2012), and the fuel characteristics of biomass are very different to those of fossil fuels (Shao *et al*., 2012). Efforts have therefore been made to understand the different fuel properties in order to optimize combustion efficiency and reduce operational problems. All biomass species will include varying degrees of N, P, K, S, Ca, Mg, Na and Si, but concentrations of K are usually considerably higher in biomass fuels compared to fossil fuels and, in grasses such as *Miscanthus*, high Cl concentrations are also common (Shao *et al*., 2012). Compared to coal, *Miscanthus* generates lower concentrations of SOx, NOx and HCl, but higher concentrations of KCl (Khodier *et al*., 2012) because of the higher concentrations of these elements, which are mainly in the form of water soluble inorganic salts that are easily volatilized during combustion. These chloride ions have a low melting temperature (<700 °C), and so form sticky layers on heat exchangers or heat transfer surfaces on boiler surfaces (Shao *et al*., 2012) and thus cause fouling, which can then inactivate catalysts (Lewandowski & Kicherer, 1997; Obernberger *et al*., 2006). Si can also be high in some *Miscanthus* genotypes (Monti *et al*., 2008), and in association with alkaline metals it can melt or sinter at 800–900 °C (Jenkins *et al*., 1998; Baxter *et al*., 2014). These alkali silicates and mixed alkali and/or calcium chlorides/sulphates tend to deposit on reactor walls or heat exchanger surfaces inside the boiler causing fouling and corrosion, even at low fusion temperatures (Wornat *et al*., 1995; Jenkins *et al*., 1998; Wang *et al*., 2012).

Various technologies have been explored to reduce the ash deposition and corrosion associated with firing/cofiring high‐alkali biomass fuels. Combustion and flue gas temperatures can be reduced during biomass thermochemical conversion (Xue *et al*., 2014), but this is energetically less efficient. Combustion systems can be designed, to some extent, to ameliorate the negative effects of elemental composition, but the accompanying increase in production and maintenance costs (slagging must be manually removed) extend operation downtime. Feedstock can be pretreated to reduce the alkali metals, and the addition of Al–Si‐based, S‐based, Ca‐based and P‐rich substances has also been explored. For example, lime and limestone have shown some effectiveness in ameliorating ash deposition and corrosion, most probably by diluting the ash, or altering the adsorption of the porous surfaces of the alkali salts when they are calcinated (rather than chemically reacted with alkali metals or alkali containing compounds). The addition of Ca‐based additives (i.e. CaO, CaCO_3_ and Ca(OH)_2_) may be effective for P‐ and K‐rich biomass fuels, by helping to convert gaseous K species into high‐melting‐point potassium silicates/phosphates, but for fuels containing high contents of K, Si and Ca, P‐based additives might be useful to reduce ash sintering and bed agglomeration (Shao *et al*., 2012). Thus, the choice of additive is dependent on the fuel type.

Another strategy, which is the subject of this study, is to optimize the biomass composition, in this case through variety selection by identifying plants with desirable flowering and/or senescence characteristics.

Despite originating from a natural cross in the wild, *M*. × *giganteus* has good combustion traits compared to other lignocellulosic crops, such as giant reed, switchgrass and sorghum (Monti *et al*., 2008; Cadoux *et al*., 2014; Iqbal & Lewandowski, 2014), and recent studies have indicated that the quality of *M*. × *giganteus* biomass can compete with wood pellets (Iqbal & Lewandowski, 2014). *Miscanthus* plants remobilize nutrients from above‐ground vegetation to storage rhizomes below‐ground at the end of the growing season. Delaying harvest from autumn, when biomass accumulation ends, to the following spring therefore results in improved quality through reduction in moisture, N, K and Cl contents. This also reduces or negates the need for fertilizer application (Beale & Long, 1997; Lewandowski & Kicherer, 1997). A negative consequence of delayed harvest is yield loss of up to 30% due to leaf drop and broken stems over the winter (Clifton‐Brown & Lewandowski, 2002; Strullu *et al*., 2011), although this helps to build up soil carbon (Mccalmont *et al*., 2015), and leaf loss improves quality as ash content is higher in this tissue type (Monti *et al*., 2008).

Plant developmental processes, such as flowering and senescence, have been postulated as important in promoting nutrient remobilization and thus improving biomass quality (Clifton‐Brown *et al*., 2001; Clifton‐Brown & Lewandowski, 2002), with senescence shown to negatively correlate with moisture content across diverse *Miscanthus* genotypes (Robson *et al*., 2012). The selection of genotypes that flower and senesce promptly and efficiently at the end of the growing season could therefore provide a strategy to create a crop: (i) with more desirable quality characteristics or (ii) that can be harvested prewinter, permitting the optimization of both yield and sustainability, and a better phasing of biomass supply to end users.

In this study, 16 genotypes (including *M. *× *giganteus*) were chosen from a population of 244 *Miscanthus* plants to represent the greatest diversity in the timing of flowering and senescence and used to understand the relationship between variation in the timing of these plant phenologies and biomass quality for N, P, K, S, Na, Cl, Si and ash contents, and heating value.

## Materials and methods

### Trial conditions

A trial of divergent *Miscanthus* germplasm was established in 2004–2005 on a sloping field (52°26′N 04°01′W) near Aberystwyth on the west coast of Wales. The soil has been classified as a Cambic stagnogley (FAO, 1998). The stone fraction (particles >2 mm) was estimated at approximately 50% of the soil mass in the 0–40 cm layer. At the time of planting (2004/5) the soil organic carbon was ca. 100 Mg ha^−1^. Climate data (temperature and radiation) were obtained from a weather station on site, whilst rainfall data were collected from a nearby weather station (52°25′N 04°01′W).

**Figure 1:**
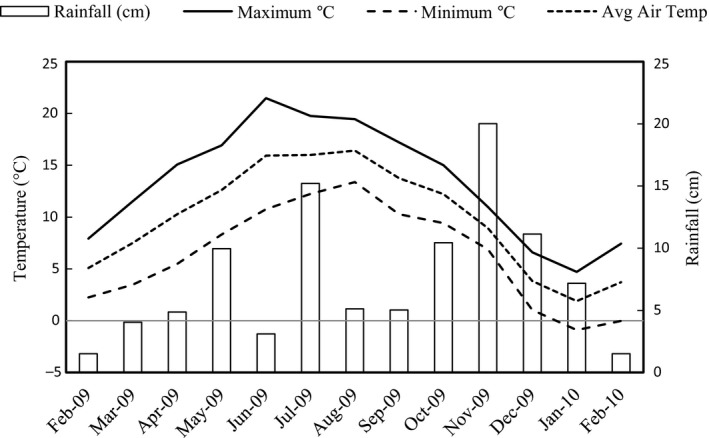
Climatic data for the trial site in Aberystwyth, Wales (52^o^25′N, 04^o^01′W), from February 2009 until February 2010.

Average monthly rainfall for 2009 (Fig. 1) was 98% of the long‐term monthly average for Gogerddan (86.5 cm). The calculated balance between rainfall and evaporation showed that mild soil moisture water deficits occurred in July, September and October in 2009, where isolated monthly values were low for rainfall and high for solar radiation (Jensen *et al*., 2011b), although these had no detectable impact on growth (based on weekly measurements of change in height of the crop canopy; data not shown). Solar radiation in 2009 was slightly higher (105%) than the long‐term average of 9.4 MJ m^−2^ day^−1^. Monthly average maximum and minimum temperatures in 2009 were similar to the long‐term mean, and soil temperatures at a depth of 5 cm did not fall below −1 °C. Once growth had started, minimum air temperatures did not drop below freezing until 1 December, and the lowest temperature reached during the experiment was −7.9 °C, in January 2010. Monthly average maximum and minimum temperatures were similar to the long‐term mean. Soil temperatures at a depth of 5 cm did not fall below –1 °C.

### Plant material

Sixteen genotypes, from a larger collection of 244, were selected to represent maximum diversity with respect to flowering and senescence (Table 1). The selection was based on the evaluation of previously recorded phenology (Jensen *et al*., 2011a; Robson *et al*., 2013a,b). Categories for flowering and senescence were allocated to each genotype (mean values for each genotype were used, *n* = 4). Flowering category was based upon the exertion of the first flag leaf on or before 21 July 2009 (early (E)), 25 August (mid (M)), after 25 August (late (L)) or not at all (non (N)). Senescence category was based upon the loss of >80% greenness of each genotype across the four replicates before 23 October (E), 24 November (M) or later (L). A number is included where more than one example was available for a particular phenotype. The genotypes were thus categorized as EE1‐3, ME1‐3, MM1, LM1‐3, LL1, NE1, NM2 and NL2 (there were no representatives for the EM, EL, ML or LE categories). Table 1 lists the categories, species and details of origin (where known).

**Table 1:** Genotype, species and details of origin [where available (NA, not available)]

Genotype	Species	Latitude	Longitude	Altitude	Country of origin
EE1	*M. sinensis*	NA	NA	NA	NA
EE2	*M. sinensis*	NA	NA	NA	NA
EE3	*M. sinensis*	38.4005	140.74	1100	Japan
ME1	Hybrid	NA	NA	NA	NA
ME2	Hybrid	NA	NA	NA	NA
ME3	Hybrid	41.9686	126.638	680	China
MM	*M. sinensis*	NA	NA	NA	NA
LM1	Hybrid	NA	NA	NA	NA
LM2	Hybrid	NA	NA	NA	NA
LM3	*M. sinensis*	38.4005	140.74	1000	Japan
LL	*M. sinensis*	34.772	132.061	200	Japan
NE1	Hybrid	35.7333	127.583	350	Korea, South
NM1	*M. sacchariflorus*	NA	NA	NA	NA
NM2	*M. sacchariflorus*	39.3567	121.866	10	China
NL1	*M. sinensis*	34.772	132.061	200	Japan
NL2	*M. sinensis*	33.3	126.583	200	Korea, South

Genotypes are classified by (a) flowering status (1st letter) and (b) senescence status (2nd letter), based on (a) the genotype exerting its first flag leaf on or before: 21 July 2009 (early (E)), between 22 July and 25 August (mid (M)), after 25 August (late (L)) or not at all (non (N)); and (b) the genotype reaching >80% loss of greenness before 23 October (E), 24 November (M) or later (L). Mean values for each genotype were used (*n* = 4). A number is included where more than one example was available for a particular phenotype.

### Design

A randomized block design was used, with four replicates. Stems were sampled from the plots on each of the harvest dates. Although this study was conducted over a single growing season, our main objectives were as follows: (i) to determine the extent of variation in mineral content in diverse *Miscanthus* genotypes and (ii) to determine the possible influence of flowering and senescence on mineral composition. Previous comparisons of flowering and senescence across 244 genotypes showed that rank orders for both traits were similar when compared across 3 years (Jensen *et al*., 2011a; Robson *et al*., 2012). Because a subset of these genotypes was used in the present study, along with a broad categorization, we were able to compare the relative performance of these genotypes in detail across a single year.

### Sample preparation and analysis

Leaf and stem material were collected on 24 June 2009 (summer), 23 October 2009 (autumn) and 11 February 2010 (spring). Stems to be harvested were chosen at random by inserting a cane marked along its length into the plant and choosing the stem closest to each mark. The number of stems chosen was based on an estimate of how much material would be required to complete elemental analyses (and therefore differed according to stem size, which differed between plants).

Moisture content was determined by taking fresh weight, and oven‐drying at 50 °C until a constant weight was reached. Residual dry matter was determined gravimetrically as the residue remaining after drying 1 g sample in a convection laboratory oven (Carbolite, Hope Valley, Hope, UK) at 102 ± 2 °C for at least 16 h. Subsequently, ash content was determined gravimetrically as the residue remaining after ignition in a muffle furnace (Carbolite) at 550 °C for at least 16 h.

N was analysed by a rapid combustion method using a LECO FP‐428 analyser (LECO Corp., St. Joseph, MI, USA) according to ISO 17025 standards. For the determination of K, Na and P, samples were prepared in accordance with AOAC (1995), MAFF (1986) and Undersander *et al*. (1993) and analyses were performed on dried and milled material. One gram of sample was weighed into 100 ml Kjeldahl tubes, 15 ml aqua regia was added and allowed to soak overnight. Samples were digested on a heating block at 120 °C for 3 h. Extracts were analysed using a Varian Liberty ICP‐AES (Agilent Technologies, Santa Clara, CA, USA).

Due to budget limitations and the expense of these types of analyses a subset of eight, from the original 16, genotypes were selected for HHV, Cl and Si analysis. Subset selection was based on representing each type of flowering and senescence classification (e.g. early, medium, late).

Determination of C, H, N, S, O, Cl and Si was carried out by MEDAC Ltd (Surrey, UK), using a FlashEA^®^ 1112 and eager300^™^ software (ThermoFisher, Waltham, MA, USA) in accordance with the operation manual (7th edition, September 2005). The laboratory is accredited to BS EN ISO9001:2008.

The calorific, or higher heating value (HHV), was determined using the equation developed by Channiwala & Parikh (2002) as: HHV = 0.3491C + 1.1783H + 0.1005S − 0.1034O − 0.0151N − 0.021 ash MJ kg^−1^.

### Statistical analysis

All statistical analyses applied to data were performed using Genstat statistical software package (17th edition; VSN International Ltd., Hemel Hempstead, UK). The analyses combined the data from all harvests. As the amount of random variation differed between harvests, a meta‐analysis was performed by fitting a linear mixed model, using the REML algorithm (Patterson & Thompson, 1971), in which a different residual variance was estimated for each harvest. Replicates within harvests were fitted as random.

The fixed model contained main effects and interactions of harvest, flowering, senescence and genotype. The terms were nonorthogonal due to the diversity in flowering and senescence over the combinations of levels of the four factors, thereby causing unequal replication of those phenologies. For example, there were no samples that had flowered or senesced at the summer harvest, and no samples that had not senesced at the autumn harvest. The order of fitting was thus important, when assessing the terms. To be sure that a factor was genuinely significant, the other factors were fitted first (i.e. *eliminated*) so that it was clear that any significant difference could not be explained by effects other than the factor of interest. Similar issues arose when assessing interaction terms. The overall effects of harvest were of less interest, so this was always fitted first. The genotype factor was always fitted last to be sure that any significance could not be explained by the varied flowering and senescence phenologies of different genotypes. There were a few genotypes at any single harvest time where some replicates had flowered and other replicates not flowered, or where some replicates had senesced and other replicates had not senesced. In these cases, there was insufficient information to be able to assess the effects of flowering or of senescence after fitting genotype. These were therefore always fitted after harvest but before genotype. Two models were assessed. In the first, flowering was fitted before senescence, and in the second senescence, it was fitted before flowering. As these developmental stages were always fitted before genotype, any conclusions about their effects are on the assumption that it is whether or not the samples had flowered (or senesced) that is the causal effect, not the distribution of the genotypes amongst the groups of flowered or nonflowered (or senesced or nonsenesced) at each of the harvests.

The assessments of the fixed terms were made using F statistics, with the denominator degrees of freedom estimated using the method of Kenward & Roger (1997). Genstat provides two tables. The first assesses the terms sequentially in the order of fitting, whilst the second assesses the effect of dropping terms from the full model. However, a term can be dropped only if there are no higher‐order terms that contain it. For example, the main effect of a factor cannot be dropped if the model contains an interaction involving that factor. Consequently, the first table was used for most of the tests reported below. The tables of predicted means use the effects from the full model, and these eliminate the effects of other terms. Consequently, they may not display the same significances as those detected using the *F*‐tests in the sequential table. Standard correlations were calculated using the predicted means from the REML analyses of the stem data.

Plots of the residuals from the analyses identified that transformations were required for some of the variables: logarithms for N, Na, K, P, Cl and Si, and logit transformations for the percentages of ash and dry matter.

## Results

Biomass samples were collected and characterized from stems and leaves, separately, of up to 16 genotypes at three harvest dates in spring, summer and autumn. We found extensive variation in all traits analysed, even in the spring harvest (February), which is when the crop would be harvested commercially for combustion. N, P, K, Si and ash contents were higher in leaves than in stems, but Cl and moisture content were higher in stems than in leaves (Tables 2, 3, 4).

**Table 2:** Predicted means (*n* = 4) and standard error for nitrogen, phosphorus and potassium content for 16 *Miscanthus* genotypes across three harvest points, at varying flowering and senescing states

	Harvest	N (mg kg^−1^)			P (mg kg^−1^)			K (mg kg^−1^)		
		Summer	Autumn	Spring	Summer	Autumn	Spring	Summer	Autumn	Spring
	Genotype	24 June	23 October	11 February	24 June	23 October	11 February	24 June	23 October	11 February
Leaf	EE1	19800 ± 0.134	7375 ± 242.82	8400 ± 393.71	2209.5 ± 191.964	1245.8 ± 244.166	1016.2 ± 81.57	9348.7 ± 347.161	1714.6 ± 180.23	1900.8 ± 425.864
	EE2	22100 ± 0.076	7450 ± 50.01	8300 ± 173.21						
	EE3	23125 ± 0.223	7200 ± 540.07	9700 ± 2657.7	2141.9 ± 288.212	2164.8 ± 628.472	1357.9 ± 165.1	7875 ± 1605.972	1284.4 ± 343.144	6459.6 ± 29.551
	ME1	18325 ± 0.08	5575 ± 421.07	6775 ± 614.25	1492.7 ± 117.979	491.4 ± 9.1	925.3 ± 133.249	8833.4 ± 1300.16	1379.5 ± 122.307	9972.9 ± 2211.795
	ME2	20800 ± 0.039	6350 ± 64.55	8075 ± 487.13	1954.2 ± 85.033	544.4 ± 46.395	1326 ± 246.523	11895.8 ± 402.02	1500.8 ± 110.13	10564.6 ± 615.93
	ME3	24200 ± 0.173	6225 ± 521.82	12667 ± 1847.83		1891.9 ± 155.426	1523.4 ± 21.551		1442.3 ± 455.253	9379.6 ± 178.5
	MM	23675 ± 0.121	11225 ± 359.11	10500 ± 922.86	2409 ± 180.463	1016 ± 77.178	2019.5 ± 326.202	9757.5 ± 718.625	3867.6 ± 93.392	14513.1 ± 1340.586
	LM1	**23125 ± 0.067**	**12325 ± 467.93**	**9000 ± 456.44**	**2390.2 ± 154.796**	**1000.3 ± 62.914**	**680.3 ± 52.385**	**11156.5 ± 92.46**	**6081.7 ± 607.265**	**1841 ± 269.074**
	LM2	22825 ± 0.035	10675 ± 478.5	8575 ± 503.95	2465.2 ± 148.874	847.6 ± 133.395	756.6 ± 136.626	12029.2 ± 678.605	4184.2 ± 669.807	2390.3 ± 702.571
	LM3	19325 ± 0.078	8400 ± 530.73	8625 ± 1418.56	1960 ± 133.226	1079.1 ± 71.485	1872.1 ± 347.432	7344.1 ± 677.304	2448.3 ± 283.005	10336.3 ± 2172.671
	LL	21825 ± 0.109	11900 ± 519.62	11750 ± 735.42	1981.6 ± 132.251	2308.4 ± 119.48	1968.1 ± 162.916	6970 ± 1515.678	3321 ± 197.121	12835.6 ± 2789.23
	NE	18433 ± 0.118	6225 ± 361.43	6575 ± 165.21	2121 ± 182.251	796.1 ± 43.815	541.5 ± 47.425	9023.9 ± 578.051	2291.7 ± 425.862	1191.6 ± 70.694
	NM1	19825 ± 0.198	12825 ± 944.62	8650 ± 520.42	2681.2 ± 214.469	1812.6 ± 317.347	764.3 ± 63.763	11277.3 ± 1691.481	5908.2 ± 1071.644	555.3 ± 45.566
	NM2	22225 ± 0.145	9550 ± 1222.37	9850 ± 753.33	2236.8 ± 78.166	1303.8 ± 163.446	1359.7 ± 51.467	6329.9 ± 736.462	2207.8 ± 730.003	5074.7 ± 352.408
	NL1	22700 ± 0.021	14625 ± 561.81	16050 ± 646.15	2229.2 ± 83.08	2336.3 ± 185.191	1295.2 ± 32.82	8847.3 ± 512.485	5361 ± 574.528	1477.4 ± 312.759
	NL2	25350 ± 0.081	13250 ± 429.15	11700 ± 470.82	2546.6 ± 161.961	2409.5 ± 158.484	1815.9 ± 167.103	9045.8 ± 1185.849	6578 ± 638.79	2915.1 ± 141.113
Stem	EE1	8400 ± 667.09	3700 ± 108.02	3375 ± 75	1306.8 ± 118.854	567.4 ± 96.772	203.3 ± 30.58	9178.3 ± 804.981	1290.3 ± 53.679	290.1 ± 22.03
	EE2	12900 ± 574.46	4875 ± 430.85	4350 ± 175.6		721.6 ± 187.207	422.2 ± 207.413		877.7 ± 91.381	314.1 ± 21.099
	EE3	7075 ± 295.46	3625 ± 232.29	3175 ± 278.02	1868.1 ± 413.361	659.4 ± 71.371	307.8 ± 16.829	760.8 ± 115.342	1298.2 ± 159.209	541.9 ± 215.176
	ME1	6050 ± 312.25	3725 ± 306.53	2950 ± 250	190.2 ± 50.711	282 ± 38.19	278.7 ± 80.372	429.8 ± 35.3	2616.8 ± 311.462	351.2 ± 54.554
	ME2	6950 ± 580.95	4300 ± 285.78	2725 ± 62.92	405.1 ± 49.171	347.3 ± 84.736	232.6 ± 16.655	708.3 ± 203.88	3163.6 ± 235.032	403.3 ± 61.399
	ME3	7575 ± 228.68	3375 ± 390.25	3025 ± 205.65	973.4 ± 193.65	387.6 ± 32.193	278.9 ± 50.236	1635.4 ± 335.389	965.5 ± 231.915	680.7 ± 264.356
	MM	9500 ± 2440.97	5350 ± 320.16	3025 ± 179.7	615.6 ± 62.333	704 ± 100.697	288.8 ± 41.064	574.3 ± 53.944	3529.7 ± 390.814	412.3 ± 60.629
	LM1	**11075 ± 1084.27**	**4225 ± 246.23**	**4000 ± 452.77**	**1408.6 ± 130.451**	**437 ± 55.933**	**378.9 ± 51.953**	**13747.7 ± 283.937**	**1973.4 ± 293.417**	**1471.7 ± 316.63**
	LM2	11250 ± 239.8	4125 ± 94.65	3725 ± 149.31	1588.7 ± 227.027	515.2 ± 87.731	330.6 ± 43.157	15497 ± 2042.022	2731.5 ± 209.372	1756.8 ± 194.654
	LM3	7575 ± 1049.91	3425 ± 426.96	2600 ± 244.95	717.6 ± 151.947	501.2 ± 44.907	365.6 ± 36.918	1288 ± 500.864	1260.6 ± 158.652	680.3 ± 185.281
	LL	7875 ± 396.61	4625 ± 131.5	5500 ± 108.02	1680.9 ± 166.411	688.3 ± 65.713	744 ± 120.781	1240 ± 337.845	2547.2 ± 349.335	3166.5 ± 1066.821
	NE	8075 ± 667.56	3300 ± 248.33	2650 ± 64.55	1399.1 ± 261.29	201.3 ± 33.419	437.5 ± 19.76	8700.9 ± 1438.312	1271 ± 228.252	324.1 ± 36.542
	NM1	10500 ± 1301.93	3700 ± 122.48	2475 ± 94.65	1744.6 ± 188.363	371.6 ± 32.948	40.8 ± 23.569	16998.2 ± 3396.482	1125.7 ± 66.325	515.1 ± 44.129
	NM2	6675 ± 990.27	3975 ± 149.31	3325 ± 170.18	453.6 ± 46.831	417 ± 92.941	222.4 ± 28.568	770 ± 114.968	1932.6 ± 633.707	1027.9 ± 312.254
	NL1	13050 ± 487.34	4825 ± 201.56	5600 ± 241.53	2071 ± 138.367	842.1 ± 70.75	1131.6 ± 137.854	17197.7 ± 1704.954	3577.9 ± 581.435	4098.2 ± 1427.481
	NL2	12200 ± 3100	6025 ± 534.44	6400 ± 408.25	1808 ± 195.4	621.4 ± 38.814	987.4 ± 36.122	12843.4 ± 1196.151	3781.4 ± 604.808	3403.1 ± 339.608

Genotype LM1 (in bold) is *Miscanthus* ×* giganteus*. Genotype categories are based on flowering and senescing phenotypes where the 1st letter denotes flowering category [exertion of first flag leaf on or before: 21 July 2009 = early (E), 25 August = mid (M)], after 25 August = late (L) or not at all = non (N); 2nd letter denotes senescence category based upon the loss of >80% greenness before: 23 October (E), 24 November (M) or later (L). Blank cells indicate insufficient material at harvest time.

**Table 3:** Predicted means (*n* = 4) and standard error for sodium, moisture content (MC) and ash and for 16 *Miscanthus* genotypes across three harvest points, at varying flowering and senescing states

	Harvest	Na (mg kg^−1^)			MC (%)			Ash % DM		
		Summer	Autumn	Spring	Summer	Autumn	Spring	Summer	Autumn	Spring
	Genotype	24 June	23 October	11 February	24 June	23 October	11 February	24 June	23 October	11 February
Leaf	EE1	180.9 ± 22.765	141.1 ± 16.744	114.4 ± 5.543	67.4 ± 0.698	43.3 ± 3.229	13.3 ± 1.017	4.6 ± 0.397	7.5 ± 0.387	5.7 ± 0.35
	EE2				65.9 ± 1.275	24.2 ± 7.308	9.5 ± 0.797	5.1 ± 0.354	6.9 ± 0.758	6.2 ± 1.141
	EE3	171.5 ± 42.206	295.1 ± 42.573	95.9 ± 20.25	69.4 ± 2.575	27.4 ± 9.661	10.6 ± 1.531	5.9 ± 0.17	12.2 ± 0.64	9.2 ± 1.077
	ME1	157.5 ± 13.143	239.4 ± 31.279	148.7 ± 17.398	67.5 ± 0.808	26 ± 5.533	9.3 ± 0.962	4.2 ± 0.313	3.7 ± 0.295	3.3 ± 0.242
	ME2	187.1 ± 13.905	262.5 ± 20.88	164.6 ± 26.969	68.1 ± 0.491	21 ± 4.039	10 ± 0.5	5 ± 0.234	5.1 ± 0.068	4.8 ± 0.362
	ME3		183.5 ± 11.031	151.3 ± 34.9	71.7 ± 3.139	25.1 ± 6.378	13.6 ± 1.649	4.6 ± 0.393	4.8 ± 0.534	3.9 ± 0.244
	MM	251 ± 24.636	317.9 ± 44.192	144.4 ± 21.255	66.3 ± 0.823	58.3 ± 0.805	11.7 ± 1.59	5.3 ± 0.463	8.2 ± 0.469	7.1 ± 0.957
	LM1	**99.8 ± 18.146**	**193.9 ± 39.728**	**159.5 ± 11.932**	**70.3 ± 1.178**	**57.3 ± 1.532**	**11.4 ± 0.885**	**5.6 ± 0.273**	**5.7 ± 0.394**	**4.8 ± 0.255**
	LM2	84.9 ± 4.115	153.1 ± 28.319	127.3 ± 14.608	69.9 ± 0.485	54.4 ± 2.606	14.2 ± 2.236	5.5 ± 0.504	6.3 ± 0.481	5.1 ± 0.221
	LM3	253.3 ± 35.774	290.7 ± 69.665	202.8 ± 32.972	68.6 ± 1.409	54.8 ± 2.123	12.7 ± 1.422	4.4 ± 0.221	5.2 ± 0.572	3.7 ± 0.208
	LL	185.6 ± 52.092	169.3 ± 14.767	205.1 ± 33.255	69.6 ± 2.061	59.3 ± 1.253	11.7 ± 0.945	4.5 ± 0.21	5.1 ± 0.171	4.5 ± 0.421
	NE	101.3 ± 1.851	188.5 ± 32.521	190.1 ± 31.517	62 ± 1.299	38 ± 5.767	11.1 ± 0.329	3.7 ± 0.23	3.2 ± 0.132	2.6 ± 0.222
	NM1	126.6 ± 36.497	166.4 ± 34.218	118.2 ± 4.824	70.3 ± 1.401	56.1 ± 2.146	9.3 ± 1.07	4.7 ± 0.341	4.7 ± 0.2	4.1 ± 0.247
	NM2	195.3 ± 19.639	210.1 ± 20.048	142.4 ± 26.221	67.9 ± 1.116	51.5 ± 3.626	12.1 ± 1.652	4.5 ± 0.116	4.5 ± 0.377	3.8 ± 0.303
	NL1	113.2 ± 5.026	136.2 ± 24.887	136.4 ± 9.277	69.5 ± 1.06	64 ± 1.342	13.4 ± 0.895	5.7 ± 0.716	5.4 ± 0.401	3.6 ± 0.409
	NL2	214 ± 91.599	174 ± 26.717	175.3 ± 23.536	72.1 ± 0.838	62.4 ± 0.845	19.2 ± 1.656	6.3 ± 0.706	5.1 ± 0.467	4.5 ± 0.588
Stem	EE1	179.9 ± 64.49	114.8 ± 7.826	77.8 ± 4.23	73.9 ± 1.379	51.9 ± 1.683	14.5 ± 1.004	3.5 ± 0.185	2.1 ± 0.044	1.4 ± 0.028
	EE2		154 ± 33.373	122.3 ± 49.834	78.5 ± 1.728	46 ± 0.626	12.1 ± 0.99	5.2 ± 0.477	2.1 ± 0.148	1.6 ± 0.188
	EE3	154.3 ± 7.512	131.4 ± 13.146	133.1 ± 16.875	79.5 ± 3.209	49.9 ± 4.662	16.9 ± 2.155	6 ± 1.443	3 ± 0.268	2.3 ± 0.343
	ME1	116.5 ± 8.236	154.7 ± 20.038	115.1 ± 8.594	74.1 ± 1.334	54.3 ± 0.63	15.5 ± 1.765	3.3 ± 0.465	1.8 ± 0.051	1.1 ± 0.101
	ME2	161.1 ± 13.646	204.5 ± 25.4	140 ± 10.517	74.1 ± 0.559	52.8 ± 0.938	14.5 ± 0.902	3.8 ± 0.28	1.9 ± 0.142	1.1 ± 0.061
	ME3	152.2 ± 6.243	125.3 ± 19.43	115 ± 20.192	81.4 ± 5.04	56.3 ± 0.466	19.8 ± 3.9	4.8 ± 1.165	1.9 ± 0.305	1.6 ± 0.15
	MM	154.4 ± 16.018	267.4 ± 72.339	135.2 ± 7.407	77.4 ± 0.963	57.3 ± 0.507	15.3 ± 1.118	5.4 ± 0.193	3.1 ± 0.117	1.7 ± 0.262
	LM1	**158.8 ± 36.234**	**199.4 ± 37.853**	**216.7 ± 8.759**	**81.5 ± 1.007**	**54.5 ± 0.247**	**27.8 ± 1.637**	**4.6 ± 0.073**	**1.6 ± 0.077**	**1.5 ± 0.048**
	LM2	154.7 ± 27.675	210 ± 9.986	222.9 ± 26.455	82 ± 0.217	55.9 ± 0.73	31.8 ± 3.622	5.1 ± 0.507	1.9 ± 0.197	1.7 ± 0.083
	LM3	139.7 ± 15.81	112.4 ± 20.548	120.9 ± 15.667	79.1 ± 1.775	60.6 ± 1.156	24.6 ± 5.369	4.4 ± 0.542	1.9 ± 0.091	1.4 ± 0.148
	LL	200 ± 8.618	164 ± 13.823	262.7 ± 66.209	81.8 ± 0.899	60.8 ± 0.914	34.3 ± 0.742	5.3 ± 0.565	2.4 ± 0.075	2.3 ± 0.096
	NE	136.6 ± 10.982	164.7 ± 16.788	61.7 ± 4.394	69.8 ± 0.597	54.5 ± 1.118	16.4 ± 0.373	3 ± 0.319	1.3 ± 0.137	0.9 ± 0.027
	NM1	415.8 ± 132.625	324.2 ± 151.665	84.1 ± 5.587	81.4 ± 1.423	58.1 ± 0.838	22.4 ± 1.309	5.2 ± 0.883	1.3 ± 0.244	0.9 ± 0.119
	NM2	150.6 ± 40.949	341.4 ± 47.361	171.7 ± 18.572	76.7 ± 1.358	64 ± 2.264	22.3 ± 1.863	3.6 ± 0.232	2.3 ± 0.194	1.5 ± 0.164
	NL1	130.6 ± 16.536	114.1 ± 10.382	132.4 ± 12.604	83.3 ± 1.056	67.5 ± 1.617	33.9 ± 2.109	7.2 ± 0.735	3.2 ± 0.418	2.9 ± 0.242
	NL2	316.8 ± 169.1	98.2 ± 2.273	94.2 ± 17.501	81 ± 0.891	60.9 ± 1.723	38.3 ± 1.426	6.3 ± 0.739	2.8 ± 0.349	2.5 ± 0.213

Genotype LM1 (in bold) is *Miscanthus* × *giganteus*. Genotype categories are based on flowering and senescing phenotypes where the 1st letter denotes flowering category [exertion of first flag leaf on or before: 21 July 2009 = early (E), 25 August = mid (M)], after 25 August = late (L) or not at all = non (N); 2nd letter denotes senescence category based upon the loss of >80% greenness before: 23 October (E), 24 November (M) or later (L). Blank cells indicate insufficient material at harvest time.

**Table 4:** Untransformed averages (mean, *n* = 4) and standard error for chlorine, silica and higher heating value (HHV) for eight *Miscanthus* genotypes across three harvest points (two for HHV) from trait trial in Aberystwyth (Wales, UK)

Harvest	Genotype	Summer	Autumn	Spring	Summer	Autumn	Spring	Summer	Autumn
		24 June	23 October	11 February	24 June	23 October	11 February	23 October	11 February
Tissue		Chlorine (mg kg^−1^)			Silica (mg kg^−1^)			HHV (MJ kg^−1^)	
Leaf	EE1	0.62 ± 0.055	0.23 ± 0.044	0.09 ± 0.033	0.44 ± 0.07	1.15 ± 0.045	1.04 ± 0.109	17.17 ± 0.116	17.35 ± 0.16
	EE2	0.26 ± 0.074	0.16 ± 0.041	0.05 ± 0	0.67 ± 0.067	1.00 ± 0.194	1.2 ± 0.255	17.32 ± 0.117	16.75 ± 0.101
	LM1	0.59 ± 0.045	0.51 ± 0.026	0.07 ± 0.014	0.35 ± 0.069	0.52 ± 0.132	0.77 ± 0.113	17.54 ± 0.125	17.58 ± 0.05
	LM2	0.61 ± 0.109	0.43 ± 0.033	0.09 ± 0.013	0.35 ± 0.047	0.73 ± 0.118	0.98 ± 0.234	17.41 ± 0.151	17.13 ± 0.081
	NE	0.30 ± 0.023	0.26 ± 0.037	0.05 ± 0	0.18 ± 0.018	0.23 ± 0.03	0.29 ± 0.044	17.53 ± 0.064	17.50 ± 0.025
	NL1	0.53 ± 0.065	0.42 ± 0.057	0.09 ± 0.015	0.62 ± 0.093	0.66 ± 0.136	0.53 ± 0.121	17.37 ± 0.053	17.69 ± 0.075
	NL2	0.59 ± 0.132	0.51 ± 0.053	0.18 ± 0.014	0.80 ± 0.162	0.46 ± 0.117	0.58 ± 0.174	17.56 ± 0.153	17.52 ± 0.111
	NM1	0.44 ± 0.056	0.37 ± 0.020	0.07 ± 0.012	0.26 ± 0.036	0.45 ± 0.074	0.59 ± 0.083	17.51 ± 0.032	17.39 ± 0.071
Stem	EE1	0.69 ± 0.046	0.19 ± 0.017	0.10 ± 0.042	0.23 ± 0.032	0.28 ± 0.02	0.21 ± 0.025	17.53 ± 0.104	17.66 ± 0.071
	EE2	1.19 ± 0.119	0.16 ± 0.033	0.11 ± 0.050	0.21 ± 0.047	0.24 ± 0.038	0.21 ± 0.069	17.17 ± 0.072	17.51 ± 0.032
	LM1	1.07 ± 0.062	0.23 ± 0.041	0.22 ± 0.018	0.15 ± 0.018	0.14 ± 0.029	0.15 ± 0.007	17.34 ± 0.116	17.69 ± 0.039
	LM2	1.19 ± 0.110	0.24 ± 0.018	0.23 ± 0.038	0.18 ± 0.016	0.19 ± 0.026	0.20 ± 0.021	17.66 ± 0.147	17.73 ± 0.049
	NE	0.60 ± 0.029	0.14 ± 0.033	0.10 ± 0.043	0.08 ± 0.005	0.13 ± 0.009	0.13 ± 0.009	17.83 ± 0.028	17.85 ± 0.038
	NL1	1.58 ± 0.226	0.39 ± 0.080	0.22 ± 0.052	0.59 ± 0.12	0.43 ± 0.072	0.40 ± 0.079	17.30 ± 0.027	17.56 ± 0.052
	NL2	1.43 ± 0.184	0.37 ± 0.064	0.18 ± 0.027	0.43 ± 0.106	0.31 ± 0.067	0.27 ± 0.069	17.41 ± 0.1	17.56 ± 0.039
	NM1	1.19 ± 0.222	0.14 ± 0.020	0.13 ± 0.033	0.11 ± 0.026	0.16 ± 0.017	0.10 ± 0.025	17.53 ± 0.013	17.87 ± 0.032

Genotype LM1 is *Miscanthus* ×* giganteus*. Genotype categories are based on flowering and senescing phenotypes where the 1st letter denotes flowering category [exertion of first flag leaf on or before: 21 July 2009 = early (E), 25 August = mid (M)], after 25 August = late (L) or not at all = non (N); 2nd letter denotes senescence category based upon the loss of >80% greenness before: 23 October (E), 24 November (M) or later (L). Blank cells indicate insufficient material at harvest time.

### Effect of harvest time and genotype on stem composition

In stems, N and K contents declined over the three harvests in 14 of the genotypes. In NL1 and 2 and LL, however, N and K declined between summer and autumn but then increased in spring, albeit to a concentration which was still lower than the summer value. P declined over the three harvests in 12 of the genotypes. In four genotypes (NL1 and 2, LL and NE), P declined between summer and autumn, but then increased in spring, again to a concentration which was still lower than in summer. Moisture content and ash declined over the three harvests in all 16 genotypes. At final harvest the stems of late‐senescing genotypes contained higher N, P, K, Si, moisture content and ash, whilst early‐senescing genotypes contained lower Cl. For example, the early‐flowering and early‐senescing genotype EE1 contained 45% of the Cl and 7% of the K contents of the genotype NL1 and contained 45% of the Cl and 19% of the K contents of LM1 (*M. *×* giganteus*).

**Figure 2:**
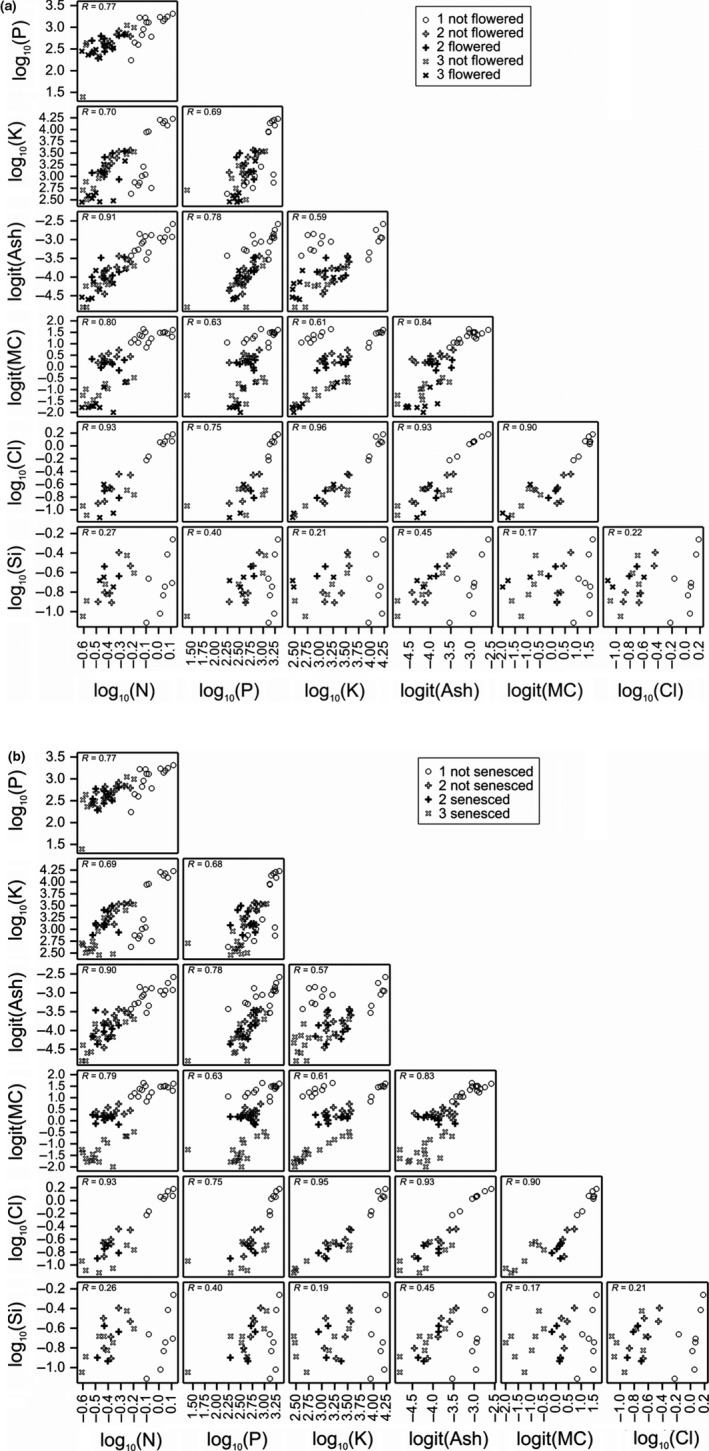
(a) Correlations between predicted means for nitrogen, phosphorus, potassium, ash, chlorine, silica and moisture content across three harvests in stems from 16 (eight for Cl and eight for Si) genotypes of *Miscanthus*. Symbols show whether the data are from the summer (o), autumn (+) or spring (×) harvests (1, 2 or 3 in key) and whether they had flowered by that harvest (in bold if flowered). (b) Correlations between predicted means for nitrogen, phosphorus, potassium, ash, chlorine, silica and moisture content across three harvests in stems from 16 (eight for Cl and eight for Si) genotypes of *Miscanthus*. Symbols show whether the data are from the summer (o), autumn (+) or spring (×) harvests (1, 2 or 3 in key). The combination of senesced and nonsenesced values was present at harvest 2 only, where senesced is indicated in bold.

Compositional traits exhibited varying degrees of association. Figure 2a, b shows the relationship between N, P, K, ash, MC and Cl using the predicted means for (i) flowering or (ii) senescence, genotype and harvest from the REML analyses of the stem data. The highest correlation (*R* = 0.96/0.95) was between Cl and K contents for flowering/senescence. Cl and K also showed correlations of >0.7 with MC (Table S2), whilst no obvious relationship was observed between Na and other variables (Table S2). Similar relationships and correlations were found using means for flowering and senescence, genotype and harvest, where the *R* values were either the same or slightly higher for flowering.

The earliest flowering occurred on 30 June, and ten genotypes initiated flowering on all four replicates, with the remainder either showing no sign of flowering or only flowered on one replicate. Compositional trait values at autumn and spring harvests (no flowering was observed at the time of the summer harvest) tended to be lower in stems that had flowered and/or senesced (Fig. 2a, b), with the exception of genotype NM1, which exhibited consistently low concentrations of N, P, K and ash (Fig. 2a, b; Table 2), despite never flowering.

In leaves, N content declined over the three harvests in four of the late (or non)‐flowering genotypes. In the other 12 genotypes, N declined between summer and autumn but then stayed constant or increased in spring, to a concentration which was still lower than in summer. K contents declined over the three harvests in five of the late (or non)‐flowering genotypes. In the other 11 genotypes, K declined between summer and autumn but then stayed constant or increased in spring, to a concentration which was still lower than in summer. P declined over the three harvests in 11 of the genotypes. In five of the genotypes, P declined between summer and autumn but then increased in spring, to a concentration which was still lower than in summer. Moisture content declined over the three harvest time points in all 16 genotypes. Ash declined over the three harvests in four genotypes. In 12 of the genotypes, ash increased or remained constant between summer and autumn, but then decreased in spring. Leaf elemental composition varied between harvest and genotype, and late‐senescing genotypes contained higher N, but the overall pattern observed in stems, where concentrations generally fell over time, was not observed in leaves (Table 2).

In both leaves and stems, significant interactions between harvest and genotype, and harvest and flowering were identified for N, P, K, Na, ash and moisture content, with some exceptions: no interaction between harvest and flowering was observed for leaf N, stem Na or leaf ash (Table 5a), and the interaction between harvest and genotype was only significant when fitted sequentially (when individual terms were dropped from the fixed model, the interaction was not significant) (Table 5a). Significant interactions were also observed between harvest and genotype for leaf Si, and leaf and stem HHV (Table 5b). Significant interactions between flowering and senescence were observed for leaf N (*P* = 0.002) and stem

**Table 5:** (a and b) *F*‐test significance values from linear mixed model

Variate	Tissue	Senescence	Flowering × senescence	Harvest × flowering	Harvest × genotype
df		1	1	1	30*
(a)					
log N (% w/w)	Leaf	<0.001	0.001	ns	<0.001
	Stem	<0.001†	ns	<0.001	<0.001
log Na (mg kg^−1^)	Leaf	ns	ns	<0.001	0.042
	Stem	ns	ns	ns	<0.001
log K (mg kg^−1^)	Leaf	<0.001	ns	<0.001	<0.001
	Stem	<0.001‡	ns	<0.001	<0.001
log P (mg kg^−1^)	Leaf	<0.001	ns	<0.001	<0.001
	Stem	<0.001	0.005	<0.001	<0.001
logit Ash % DM	Leaf	<0.001	ns	ns	<0.001
	Stem	<0.001	ns	<0.001	0.014
logit MC % DM	Leaf	<0.001	ns	0.035	<0.001
	Stem	<0.001	ns	<0.001	<0.001

Variate	Tissue	Harvest	Flowering	Senescence	Genotype	Harvest × genotype
df		2§	1	1	7¶	13**
(b)						
log Cl (% w/w)	Leaf	<0.001	<0.001	<0.001	<0.001	ns
	Stem	<0.001	0.011††	0.004‡‡	<0.001	ns
log Si (% w/w)	Leaf	<0.001	<0.001	0.012§§	<0.001	0.004
	Stem	ns	0.003¶¶	0.010§§	<0.001	ns
HHV (MJ kg^−1^)	Leaf	0.046	<0.001	ns	0.013	0.01
	Stem	<0.001	0.004***	ns	<0.001	ns

^*^df 28 for Na, K and P leaf, and 29 for Na, K and P stem.

^†^If fitted before flowering, otherwise 0.001.

^‡^If fitted before flowering, otherwise 0.034.

^§^df 1 for HHV.

^¶^df 6 for HHV.

^**^df 6 for HHV.

^††^Only when fitted before senescence, otherwise ns.

^‡‡^If fitted after senescence, otherwise 0.039.

^§§^If fitted after flowering, otherwise ns.

^¶¶^If fitted after senescence, otherwise 0.041.

^***^If fitted before senescence, otherwise 0.005.

P (*P* = 0.005). No interactions were observed between flowering and genotype or senescence and genotype, or between flowering, harvest and genotype. However, a three‐way table of predicted means is presented for N, P, K, Na, ash and moisture contents due to the significant interactions involving combinations of harvest with genotype and harvest with flowering (Table S1). Figure S1 illustrates the genotypic trends and interactions with harvests for stem N, P and K.

Only 10 samples (comprising five genotypes) exhibited detectable (>0.1%) concentrations of S. These were almost all (9/10) in spring harvested leaf material and half of these were from a single genotype (NL2).

As the majority of the harvested *Miscanthus* biomass is stem, we present more detailed findings for this fraction only. A closer examination of the key traits follows, and includes an analysis of the effects of flowering and senescence.

### Nitrogen

Mean stem N concentrations in the spring were 0.37% w/w (minimum 0.2% w/w), which was less than half the concentrations at the summer harvest (mean 0.93% w/w, minimum 0.6% w/w) (Table 2). The seds for the genotype x harvest interaction and predicted mean values (Tables 2 and S1) indicated that these differences were highly significant from summer to autumn in every genotype, but from autumn to the following spring were not significant in seven of the genotypes (EE1, 2 and 3, LM1 and 2, LL, NL1 and 2). The difference in (transformed) N concentrations between flowered and nonflowered stem material was highly significant in the spring, but not in the autumn (Table 6). The difference between (back‐transformed) senesced and nonsenesced stem N concentrations was 0.27%, which was also highly significant (Table 6).

**Table 6:** Differences between predicted means

Variate	Senescence (main effects)				Flowering differences in autumn (harvest 2)				Flowering differences in spring (harvest 3)		
	df	Difference†		SED	df	Difference		SED	Difference		SED
		Transformed	Back‐transformed			Transformed	Back‐transformed		Transformed	Back‐transformed	
log N (% w/w)	42.1	0.244***	0.02698	0.01116	67.1	0.0228	0.0208	0.01693	0.0447**	0.0362	0.01543
log P (mg kg^−1^)	40.5	0.0336***	401.3	0.0384	82.2	−0.46	−51.2	0.03536	0.048	35.5	0.05299
log K (mg kg^−1^)	18	0.391***	1397	0.02355	31.3	0.045	174	0.04088	0.366***	670	0.05685
log Na (mg kg^−1^)	42.8	0.078	26.4	0.02454	60.2	0.05	17.8	0.04359	0.001	0.4	0.03441
logit Ash % DM	42.9	0.743***	1.687	0.03629	65.5	−0.172	−0.348	0.06291	0.059	0.087	0.05725
logit MC % DM	41.6	1.753***	41.22	0.03684	88.2	0.2531***	6.23	0.03996	0.5464***	9.29	0.0694

**indicates significance at 0.01% and ***indicates significance at 0.001%, for a 2 tail test.

†Difference between senesced and nonsenesced.

In three cases (NL1 and 2, LL), N concentrations were higher in the spring compared with autumn, but these were amongst the genotypes whose spring N content was not significantly different from that in autumn (Table S1). No relationship between starting concentration of N and subsequent concentrations was identified: genotypes with the highest initial concentrations did not necessarily have the highest concentrations throughout the harvests.

### Phosphorous

Mean stem P concentrations in the spring were 416 mg kg^−1^ (minimum 41 mg kg^−1^), which was, again, less than half the concentration in the previous summer (mean 1215 mg kg^−1^, minimum 190 mg kg^−1^). Five exceptions to the reduction of P concentrations throughout the harvests were identified (Table 2), and there were nine genotypes (ME1, 2 and 3, LM1, 2 and 3, LL, and NL1 and 2) whose spring P content was not significantly different from that in the autumn (Tables 2, 6 and S1). In one case (ME1), P concentrations were well below the average at each harvest point. The differences between P concentrations in nonflowered and flowered stems in the autumn and spring were not significant (Table 6).

### Potassium

Across 16 genotypes analysed across three harvest points we found the greatest variance to be in stem K concentrations between the summer and spring harvests, with a minimum and maximum value of 290 and 17 197 mg kg^−1^, respectively (EE1 in spring and NL1 in summer, respectively (Table 2). In contrast to N, where concentrations were consistently highest in the summer, for seven genotypes K concentrations were highest in the autumn (Table 2). K concentrations fell again by the following spring in all but three genotypes (LL, NL1 and NL2) which also exhibited anomalous patterns of N concentration. With the exception of the EE phenotypes, the same genotypes that did not show significantly different N content in spring compared with autumn also did not show significantly different K content (LM1 and 2, LL, NL1 and 2). The difference in K concentrations between flowered and nonflowered stems in the spring was 670 mg kg^−1^ (untransformed), which was highly significant (Table 6). Similarly, the difference between senesced and nonsenesced stems was 1397 mg kg^−1^ (untransformed), which again was highly significant (Table 6).

### Moisture content

Stem moisture content fell significantly with each successive harvest in every genotype (Table 3). The lowest moisture contents in both the autumn (mean 57%, minimum 46%) and spring (mean 22%, minimum 12%) were in the earliest flowering and senescing genotypes (Table 3), and differences between (untransformed) nonflowered and flowered stem moisture contents were 6.2% in the spring and 9.3% in the autumn, which were both highly significant (*P* < 0.001 with 88.2 df; Table 6).

### Ash

Stem ash contents also fell with successive harvests (Table 3) and although these were significant in each genotype from summer to autumn, ash contents in spring were not significantly different from autumn content in the same five genotypes (LM1 and 2, LL, NL1 and 2) that had nonsignificant N and K concentrations in spring compared to autumn. Ash contents in nonflowered stems in the autumn were lower than in flowered stems, and vice versa in the spring, although neither of these differences were significant (Table 6).

### Chlorine

Stem Cl concentrations fell fivefold from summer to autumn (1.05 and 0.21% w/w, respectively, *P* < 0.001) and again from autumn to spring (0.21–0.14% w/w, respectively, *P* < 0.01). Stems that had not flowered contained more than twice (*P* < 0.001; Table 7) the Cl of those which had flowered (0.36 and 0.15% w/w, respectively), and stems that had not senesced contained more than three times (*P* < 0.001; Table 7) the Cl of those stems which had senesced (0.56 and 0.15% w/w, respectively). There were also highly significant differences between genotypes, and the most extreme differences are reported in Table 7 for illustration purposes. The lowest concentrations of Cl in the autumn and spring were 0.14 (genotype NE) and 0.10 (genotype NE) % w/w, respectively, compared to the highest concentrations of 0.39 (genotype NL1) and 0.23 (genotype LM2) % w/w, respectively (Table 4).

**Table 7:** Main effects for chlorine and silica

Variate	Harvest					Flowering			Senescence			Genotype		
	df	Difference†	SED	Difference‡	SED	df	Difference	SED	df	Difference	SED	df	Difference§	SED
Chlorine	28.1	0.7058***	0.03995	0.1587**	0.05885	48.3	0.3888***	0.06649	22.1	0.5749***	0.0425	35.3	0.418¶	0.0737
Silica	15.3	−0.0325	0.04101	0.0454	0.04209	60.8	−0.1032*	0.04812	19.7	0.0572	0.03779	55.7	0.6075	0.0640

*Indicates significance at 0.1%; **indicates significance at 0.01% and ***indicates significance at 0.001%, for a 2 tail test.

^†^Difference from spring to autumn.

^‡^Difference from autumn to spring.

^§^Differences between highest and lowest values.

^¶^For some genotypes.

### Silica

Stem Si concentrations were much less variable than other compositional traits analysed and only varied significantly from autumn to spring in genotypes EE1 and NM1. Neither were they significantly affected by senescence (Tables 5b and 7). However, flowering appeared to slightly increase (*P* = 0.05) stem Si (Tables 5b and 7). There were also highly significant differences between genotypes: the lowest concentrations of Si in the autumn and spring were 0.13 (genotype NE) and 0.10 (genotype NM1) %, respectively, compared to the highest concentrations of 0.43 (genotype NL1) and 0.40 (genotype NL1) %, respectively (Table 4). Initial correlation analyses showed little relationship between Si content and any of the other variables. However, the pattern of Si correlations shows a shift to the right for the summer harvest data with every variable (see Fig. 2a, b). When the correlations were performed separately for summer, autumn and spring, Si presented correlations with ash of 0.741, 0.897 and 0.921, respectively, the latter being the highest correlation of the entire dataset.

### HHV

The increase in mean HHV between autumn (17.5 MJ kg^−1^) and spring (17.69 MJ kg^−1^) was highly significant (*P* < 0.001), as were the differences between genotypes (*P* < 0.001). The table of fixed effects, where flowering is fitted before genotype, suggests that flowering significantly impacts HHV, but when the full model is taken into account the difference between flowered and nonflowered stems is only 0.08 (SED of 0.04978, df 14.5), which is not significant. The minimum HHV exceeded 17 MJ kg^−1^ in each genotype and in both autumn and spring harvests.

## Discussion

### Biomass quality improvement through delayed harvest is genotype‐specific

Despite yield losses of up to 30%, standard agronomic practice for commercial *Miscanthus* biomass production is to delay harvest until the spring following growth in order to allow improvement to combustion quality through moisture loss, nutrient remobilization and leaching of soluble minerals, like K and Cl, through rainfall (Jorgensen, 1997; Lewandowski & Kicherer, 1997). This practice also helps ensure crop sustainability, as nutrients will not need replacing for subsequent growth. However, the composition of *M*. × *giganteus* in our study (genotype LM1), which was comparable to values reported elsewhere (Clifton‐Brown & Lewandowski, 2002; Lewandowski & Heinz, 2003; Monti *et al*., 2008; Meehan *et al*., 2013; Yu *et al*., 2014), did not significantly change between October and the following February for a number of key parameters. Indeed, our REML analysis of 16 genotypes showed that five of these (including *M. *× *giganteus*) did not exhibit significant reduction in N, P, K and ash from autumn (October) to winter (February), and two of these (including *M. *× *giganteus*) also did not show significantly reduced Cl and Si content from autumn to spring. A sixth genotype (NM1), despite showing improved quality from autumn to spring, exhibited sufficiently low N and K content in autumn to meet the EN*plus* wood pellet standards for those traits (European Pellet Council, 2015). All genotypes exceeded the ≥16.5 MJ kg^−1^ requirement for EN*plus* wood pellet certification, even in the autumn (Table 4), and HHV increased significantly from autumn to spring in only half the genotypes tested. Moreover, stem Si concentrations were consistent across the year, with minimums/maximums of 0.11%/0.59%, 0.13%/0.43% and 0.10%/0.40% in summer, autumn and spring, respectively (Table 4). On the other hand average leaf Si concentrations increased significantly from summer (0.46%) to autumn (0.65%), with a lesser increase again from autumn to spring (0.75%). There was a fivefold difference between the minimum (0.23%) and maximum (1.15%) concentrations of Si in the autumn. With respect to Si, therefore, there was no benefit, and even some detriment, in delaying harvest until spring.

Average leaf and stem N concentrations in the autumn were 43% and 46%, respectively, of summer values, but surprisingly, from autumn to spring the overall average leaf N content increased very slightly, whilst stem N content decreased by only 13% of autumn values (Table 2), and the decrease in stem N from autumn to spring was significant in only half the genotypes tested. This lack of significant N reduction over winter was consistent with findings by Jorgensen (1997), who suggested that N must therefore be fixed in nonsoluble organic substances. However, others have reported significant N content reduction over winter months (Lewandowski *et al*., 2003; Baxter *et al*., 2014), which may be due to differences in N fertilizer regimes: there was no fertilizer treatment in our trial.

For N and ash content in stem material, we identified four genotypes (ME1, ME2, NM1 and NM2) that met the EN*plus* A2 pellet classification criteria when harvested in spring, two of which were close to reaching A1 standards (European Pellet Council, 2015). Furthermore, two of the aforementioned genotypes, NM1 and NM2, were within N concentration limits for the A2 classification during the autumn harvest in October, and only 0.1% over the ash limit. This is promising for an autumn harvest at peak yield, although the leaf fraction would need to be minimized.

Consistent with previous studies (Jorgensen, 1997; Lewandowski *et al*., 2003; Baxter *et al*., 2014) leaf N concentrations were much higher than those of stem, and during the spring, they were comparable (even slightly higher) than those of stems in the summer. This illustrates the importance of leaf loss over the winter, which varies greatly between *Miscanthus* genotypes (Lewandowski *et al*., 2003), and should be considered a key trait for the optimization of biomass quality because, with the sole exception of spring Cl content, mineral concentrations were overall lower in stem compared with leaf material at each harvest (Tables 2, 3, 4), consistent with previous reports (Lewandowski *et al*., 2003; Monti *et al*., 2008; Baxter *et al*., 2014).

### Flowering and senescence as factors affecting *Miscanthus* biomass quality

The relationship between flowering and senescence in *Miscanthus* has not been well defined but it has been proposed that both potentially promote nutrient remobilization, and hence combustion quality and sustainability. We did not identify an unambiguous correlation (*R* = 0.52) between flag leaf emergence (the first visible sign of flowering) and senescence; however, the order of fitting these terms in our model influenced the level of significance for some traits (Table 5a and b). Genotypes had been selected in our study that exhibited variation in flowering time and senescence to explore the effect of these phenologies. We did not physically control flowering and senescence in this experiment. Nevertheless, for N, P, K, Cl, ash and moisture there was a clear reduction in contents and therefore improvement in quality associated with senescence, and a less pronounced but still significant improvement to N, K, Cl (for spring harvest only) and moisture content (for autumn and spring harvests) associated with flowering (Table 5a and b). However, despite these overall trends, the very late‐flowering (did not flower at all in our trial) and medium‐senescing genotype NM1 exhibited an exceptional phenotype for chemical combustion, with consistently low concentrations for all compositional traits, and in the case of N, P and Si, the lowest concentrations of all genotypes examined. This genotype also exhibited the highest heating value and we therefore suggest would make an excellent genotype for *Miscanthus* variety improvement.

We speculated that stems that had flowered and were senescing by autumn were more prone to Cl (and K) leaching and thus had improved quality by spring (Jorgensen, 1997). We also expected that leaching might be promoted in thinner‐stemmed genotypes. However, genotype NM1 confounded both of these theories as it did not flower at all in our trial, but had the lowest overall mineral content, and it also had the thickest stems of all the genotypes in our trial (15.4 cm; Table S3). We also compared the progression of senescence in this medium‐senescing genotype with those of early‐senescing types (Fig. S2), expecting to see a prompt completion of senescence before the autumn harvest that explained its performance. However, this genotype had reached <60% loss of greenness by the October harvest and yet had lower N and K contents than some of the early‐senescing genotypes (Table 2) and the lowest Cl and ash contents of all genotypes at that harvest.

In the case of LM1, LM2 and NM1, stem Cl concentrations barely declined between autumn and spring, whereas contents decreased consistently and significantly in the remaining five genotypes analysed (Table 4). Jorgensen (1997) identified reductions of about 50% in Cl content of *M. *× *giganteus* over winter, but 85–95% in two clones of *M. sinensis*, which could not, therefore, be attributed to weather patterns. Jorgensen (1997) highlights the role of frost damage in promoting leaching, and that cuticle integrity, leaf age and tissue wettability, as well as membrane malfunction, are known to affect leaching (Charley & Richards, 1983). Jorgensen (1997) also highlights the range in frost sensitivity of (i) different plants, where membrane function may be retained or lost only transiently after frost in a frost‐hardy plant, and any ions diffused from the cell would be reabsorbed after thawing; and (ii) different tissues, where studies in sugar cane show that lethal temperatures range from −2.8 °C for leaves of young plants, and −5.0 to −5.6 °C for lower stems. *M*. ×* giganteus* and late‐ripening selections of *M. sinensis* were observed as apparently alive during part of the winter, even in Denmark (Jorgensen, 1997), where some tissues are not killed until severe frosts appear. In our study, the coldest temperature was −7.94 °C, on 8 January. Substantial leaching may therefore not occur in some genotypes until lethal frost temperatures occur, and certainly many *Miscanthus* genotypes are far more frost‐hardy than sugar cane. In our trial, the three stay green *M. sinensis* genotypes (LL, NL1/2) showed much higher levels of K (as well as N, P, ash and MC) in the spring than other genotypes, and similar Cl content to *M. *× *giganteus*. One of these, NL2, also contained the highest concentrations of Si and S and is thus highly unsuitable for breeding, but could make a valuable parent in a cross to help dissect senescence and composition traits.

A further explanation for confounding mineral content could be due to influx and efflux of different elements. In cereal leaves, chloroplasts contain 75% of the reduced N in the cell (Peoples & Dalling, 1978). The remobilization of minerals affected by senescence has been less well researched, but studies in trees (Walter *et al*., 1988) demonstrated the translocation of minerals, both in and out of leaf blades, during autumn senescence. The complex interactions of senescence processes may explain why some mineral elements declined rapidly, for example N and K from summer to autumn, and others, such as Cl, were maintained at a more steady level in our study. The large translocations of nutrients no longer required in above‐ground tissues, particularly with elements such as N, result in a net decrease in biomass within those tissues; as a consequence other minerals, when measured as a proportion of biomass, they may remain at relatively high levels despite active translocation occurring, because their efflux is masked by larger changes in other minerals and biomass yield.

Such complex translocations may also affect N levels. Detailed comparisons of the visual progression of senescence showed that the majority of early‐senescing genotypes were above an 80% loss of greenness before the autumn harvest, and some had even completed senescence by then (Fig. S2). Therefore, the major translocation of N would be expected to be completed in these genotypes. Where N is appearing to increase slightly in leaf tissue between autumn and winter harvests is possibly the result of an efflux of other minerals from leaf tissues and a general overall decline in biomass, resulting in the proportion of N in the remaining biomass appearing to increase.

### Agronomic practices in *Miscanthus* quality improvement

Despite the favourable N and ash contents reported above that met current wood pellet standards, the lowest Cl concentration we identified (0.1%) was approximately threefold higher than the acceptable EN*plus* limit of ≤0.03 for A2 classification wood pellets. Cl content in grasses is known to be higher than in wood, although *Miscanthus* Cl content is favourable to those of cereal straw (0.4%) and reed canary grass (0.6%) (Obernberger *et al*., 2006). Cl concentrations in the present study were comparable to those of Jorgensen (1997) and Meehan *et al*. (2013), but exceeded those of Baxter *et al*. (2014). However, Jorgensen (1997) found interannual differences of 0.32% Cl in *M. *×* giganteus*, and it is possible that *Miscanthus* grown at coastal locations, as in the present study, may have elevated Cl due to salt spray. Importantly however, 80–90% of Cl (as well as K) has been shown to leach in mown barley straw exposed to 100 mm precipitation (Sander, 1997), and more recently, it was found that cutting *M. *×* giganteus* in January, but leaving it in the field prior to collection in April, led to a fall of ~0.4% in Cl content compared to that cut and harvested in April. This was considered a likely consequence of leaching due to rainfall (Meehan *et al*., 2013). Leaving the crop on the field postharvest could therefore vastly improve quality, especially in climates like Wales where the greatest rainfall (~200 mm) tends to be in November (e.g. as in Fig. 1).

Although EN*plus* is the benchmark for wood pellets, in recent years standards have been emerging for straw and grass fuel pellets (Carroll & Finnan, 2012; Miranda *et al*., 2015). As markets develop, greater value will be placed on quality, and meeting these standards will require management of quality throughout the supply chain from grower through to end user. This will include variety selection and agronomy over the feedstock production cycle to optimize combustion efficiency, minimize slagging and fouling and meet emissions targets. Plant breeding is thus an important aspect of achieving quality targets as an alternative, or in addition, to pretreatment processes such as pellet washing/leaching with water (Jenkins *et al*., 1998; Yu *et al*., 2014), surfactants (Banks *et al*., 2014), acid or other solutions (Saddawi *et al*., 2012). Indeed, the advantages of early and/or rapid senescence is likely to be that resources are remobilized before an early harvest to the rhizome and that winter rainfall will promote the leaching of elements such as K and Cl from the standing stems. Additional leaching could be achieved by cutting stems and leaving flat or in swaths in the field (Meehan *et al*., 2013). This may be particularly important in achieving potentially rigorous Cl standards and will also help reduce MC % (Meehan *et al*., 2013).

Quantitative trait loci (QTL) investigations aimed at identifying regions of the *Miscanthus* genome that impact on combustion traits have been reported (all in *M. sinensis*). These were conducted on a population of limited size (*n* = 89), and an incomplete genetic map (comprising 28 linkage groups, whilst *M. sinensis* has 19 chromosomes). The linkage groups were identified using RAPD markers, which have limited reproducibility (Atienza *et al*., 2002, 2003a,b,c,d). We propose the development of new mapping families using material similar to that identified as NM1 in the present study for the purposes of improving combustion traits and promoting sustainability. There also may be a need to develop mapping families for the identification of traits relating to biomass quality for varied end uses, that is combustion, pyrolysis and gasification or fermentation.

## Conclusions and recommendations

- Significant variations in *Miscanthus* mineral content between genotypes and harvest times were found under the environmental conditions at a site near Aberystwyth, Wales, UK. These are being used to guide parental selections needed to make breeding improvements in the compositional characteristics that influence biomass quality.
- Not all quality relevant traits were significantly improved by delaying harvest until spring (February), when harvestable yields determined in plot trials are typically one‐third less than in late autumn (October). Five (including *M. *× *giganteus*) of sixteen genotypes did not exhibit the expected significant overwinter reductions in N, P and K. In two of these genotypes (including *M. *× *giganteus*), there was also no significant overwinter reduction in Cl.
- Early flowering and senescence were not closely correlated with each other, but both were shown to impact mineral content, thus improving biomass quality attributes generally. Exceptionally, one late‐flowering genotype that does not flower or senesce thoroughly in Aberystwyth during winter (NM1) exhibited excellent overall quality traits, with sufficiently low N and K content in autumn to meet the EN*plus* wood pellet standards (European Pellet Council, 2015).
- Whilst breeding should select parents with optimized quality traits, agronomic practices should also be optimized, such as avoiding overfertilization, identifying the most appropriate harvest time, and cutting plants before collection to allow maximum moisture loss and leaching of water soluble minerals.
- Few genotypes in our trial had <50% MC at the October harvest. However, Lewandowski & Heinz (2003) concluded that for CO_2_ equivalents, harvesting *Miscanthus* in December, before winter losses, resulted in higher hectare‐related CO_2_ equivalent saving potentials than a February or March harvest, despite the increased energy demand for technical drying.
- Additional studies in changes to the chemical composition of *Miscanthus* biomass over the growing season across diverse locations and years are recommended to determine consistencies in nutrient dynamics and enable valuable modelling for varied soils and environments, and thus end uses.

## Supporting information


**Table S1.** Predicted means for N, P, K, Na, ash, and MC from REML Genotype and Harvest analysis at available flowering states (flowered vs. non‐flowered) for diverse *Miscanthus* gentoypes from trait trial in Aberystwyth (Wales, UK). Genotype LM1 is *Miscanthus* ×* giganteus*.
**Table S2.** Correlation matrices between nitrogen, sodium, potassium, phosphorus, chlorine, silica, moisture content, ash and higher heating value (HHV) based on flowering (a) and senescence (b), genotype and harvest from the REML analyses of stem data for diverse *Miscanthus* genotypes across three harvest points (summer, autumn and spring) in 2009–2010 as part of trait trial in Aberystwyth (Wales, UK).
**Table S3.** Plant characteristics of the 16 *Miscanthus* genotypes (two species and their hybrids) studied, including: plant basal diameter (mm), transect count (a relative measure of stem number), height of tallest stem (mm), canopy height (mm) and dry matter single plant biomass (g).
**Figure S1.** Genotypic trends and interactions with harvest for stem N, P, and K in diverse *Miscanthus* genotypes.
**Figure S2.** Senescence progression (recorded as loss of greenness, where 10 = 100%) in early senescing genotypes, and genotype NM1, of *Miscanthus* grown in 2009 trait trial in Aberystwyth (Wales, UK).
