# Supplementary material for: Towards Miscanthus combustion quality improvement: the role of flowering and senescence
Source: Glob Change Biol Bioenergy. 2016 Sep 30;9(5):891–908. doi: 10.1111/gcbb.12391 (PMC5412913; doi:10.1111/gcbb.12391)
Supplement: Supplementary file 1 — Table S1. Predicted means for N, P, K, Na, ash, and MC from REML Genotype and Harvest analysis at available flowering states (flowered vs. non‐flowered) for diverse Miscanthus gentoypes from trait trial in Aberystwyth (Wales, UK). Genotype LM1 is Miscanthus × giganteus. Table S2. Correlation matrices between nitrogen, sodium, potassium, phosphorus, chlorine, silica, moisture content, ash and higher heating value (HHV) based on flowering (a) and senescence (b), genotype and harvest from the REML analyses of stem data for diverse Miscanthus genotypes across three harvest points (summer, autumn and spring) in 2009–2010 as part of trait trial in Aberystwyth (Wales, UK). Table S3. Plant characteristics of the 16 Miscanthus genotypes (two species and their hybrids) studied, including: plant basal diameter (mm), transect count (a relative measure of stem number), height of tallest stem (mm), canopy height (mm) and dry matter single plant biomass (g). Figure S1. Genotypic trends and interactions with harvest for stem N, P, and K in diverse Miscanthus genotypes. Figure S2. Senescence progression (recorded as loss of greenness, where 10 = 100%) in early senescing genotypes, and genotype NM1, of Miscanthus grown in 2009 trait trial in Aberystwyth (Wales, UK). [file GCBB-9-891-s001.docx]

Supplementary Table 1. Predicted means for N, P, K, Na, ash, and MC from REML Genotype and Harvest analysis at available flowering states (flowered vs non-flowered) for diverse *Miscanthus* gentoypes from trait trial in Aberystwyth (Wales, UK). Genotype LM1 is *Miscanthus* *x giganteus*. Genotype categories are based on flowering and senescing phenotypes where the 1^st^ letter denotes flowering category (exertion of first flag leaf on or before: 21^st^ July 2009=early (E); 25^th^ August = mid (M)); after 25^th^ August = late (L), or not at all = non (N); 2^nd^ letter denotes senescence category based upon the loss of >80% greenness before: 23^rd^ October (E); 24^th^ November (M); or later (L). Blank cells indicate insufficient material at harvest time.

Shaded cells indicate values pertaining to plants that had flowered, italicised cells indicate values are for non-flowered plants but where flowered data also exist in one or more of the four replicates, in which case these values are presented on a line underneath and shaded.

| Supplementary Table 2. Correlation matrices between nitrogen, sodium, potassium, phosphorus, chlorine, silica, moisture content, ash and higher heating value (HHV) based on flowering (a) and senescence (b), genotype and harvest from the REML analyses of stem data for diverse *Miscanthus* genotypes across three harvest points (summer, autumn and spring) in 2009-2010 as part of trait trial in Aberystwyth (Wales, UK). | | | | | | | | | | |
| --- | --- | --- | --- | --- | --- | --- | --- | --- | --- | --- |
|  |  |  |  |  |  |  |  |  |  |  |
| (a) | **N** | 1 |  |  |  |  |  |  |  |  |
|  | **Na** | 0.08 | 1 |  |  |  |  |  |  |  |
|  | **K** | 0.686 | 0.407 | 1 |  |  |  |  |  |  |
|  | **P** | 0.77 | 0.119 | 0.565 | 1 |  |  |  |  |  |
|  | **Cl** | 0.561 | 0.425 | 0.896 | 0.47 | 1 |  |  |  |  |
|  | **Si** | 0.719 | -0.101 | 0.521 | 0.734 | 0.528 | 1 |  |  |  |
|  | **MC** | 0.405 | 0.396 | 0.74 | 0.386 | 0.713 | 0.307 | 1 |  |  |
|  | **Ash** | 0.883 | 0.095 | 0.754 | 0.757 | 0.735 | 0.892 | 0.513 | 1 |  |
|  | **HHV** | -0.681 | -0.047 | -0.323 | -0.566 | -0.389 | -0.535 | -0.5 | -0.654 | 1 |
|  |  | **N** | **Na** | **K** | **P** | **Cl** | **Si** | **MC** | **Ash** | **HHV** |
|  |  |  |  |  |  |  |  |  |  |  |
|  |  |  |  |  |  |  |  |  |  |  |
|  |  |  |  |  |  |  |  |  |  |  |
|  |  |  |  |  |  |  |  |  |  |  |
| (b) | **N** | 1 |  |  |  |  |  |  |  |  |
|  | **Na** | 0.122 | 1 |  |  |  |  |  |  |  |
|  | **K** | 0.686 | 0.429 | 1 |  |  |  |  |  |  |
|  | **P** | 0.756 | 0.13 | 0.566 | 1 |  |  |  |  |  |
|  | **Cl** | 0.569 | 0.395 | 0.891 | 0.488 | 1 |  |  |  |  |
|  | **Si** | 0.666 | -0.166 | 0.456 | 0.706 | 0.507 | 1 |  |  |  |
|  | **MC** | 0.372 | 0.445 | 0.754 | 0.399 | 0.756 | 0.308 | 1 |  |  |
|  | **Ash** | 0.857 | 0.08 | 0.731 | 0.754 | 0.74 | 0.884 | 0.523 | 1 |  |
|  | **HHV** | -0.655 | -0.104 | -0.323 | -0.557 | -0.405 | -0.536 | -0.446 | -0.652 | 1 |
|  |  | **N** | **Na** | **K** | **P** | **Cl** | **Si** | **MC** | **Ash** | **HHV** |

Supplementary Table 3. Plant characteristics of the 16 *Miscanthus* genotypes (two species and their hybrids) studied, including: plant basal diameter (mm), transect count (a relative measure of stem number), height of tallest stem (mm), canopy height (mm) and dry matter single plant biomass (g). Genotype categories are based on flowering and senescing phenotypes where the 1^st^ letter denotes flowering category (exertion of first flag leaf on or before: 21^st^ July 2009=early (E); 25^th^ August = mid (M)); after 25^th^ August = late (L), or not at all = non (N); 2^nd^ letter denotes senescence category based upon the loss of >80% greenness before: 23^rd^ October (E); 24^th^ November (M); or later (L).

| Genotype | Species | Plant basal diameter | Transect count | Tallest stem height | Max canopy height | Stem diameter | Dry matter biomass |
| --- | --- | --- | --- | --- | --- | --- | --- |
| EE1 | *M. sinensis* | 363 | 35 | 1910 | 1175 | 4.4 | 785 |
| EE2 | *M. sinensis* | 263 | 35 | 1340 | 1000 | 3.1 | 307 |
| EE3 | *M. sinensis* | 438 | 38 | 1590 | 988 | 4.1 | 597 |
| ME1 | Hybrid | 488 | 31 | 2130 | 1513 | 4.6 | 1017 |
| ME2 | Hybrid | 563 | 39 | 1960 | 1563 | 4.0 | 1468 |
| ME3 | Hybrid | 325 | 27 | 1045 | 938 | 4.0 | 261 |
| MM1 | *M. sinensis* | 500 | 36 | 2035 | 1613 | 4.8 | 1438 |
| LM1 | Hybrid | 638 | 22 | 2450 | 2625 | 7.3 | 3773 |
| LM2 | Hybrid | 525 | 17 | 2435 | 2550 | 8.2 | 2001 |
| LM3 | *M. sinensis* | 488 | 27 | 2000 | 1750 | 6.4 | 1900 |
| LL | *M. sinensis* | 450 | 29 | 1718 | 1575 | 5.7 | 1940 |
| NE | Hybrid | 513 | 32 | 1410 | 1688 | 3.8 | 534 |
| NM1 | *M. sacchariflorus* | 1163 | 35 | 1533 | 1825 | 15.4 | 1363 |
| NM2 | *M. sacchariflorus* | 738 | 21 | 898 | 1025 | 3.3 | 218 |
| NL1 | *M. sinensis* | 425 | 26 | 1355 | 1700 | 5.5 | 1506 |
| NL2 | *M. sinensis* | 338 | 17 | 805 | 1075 | 6.1 | 898 |


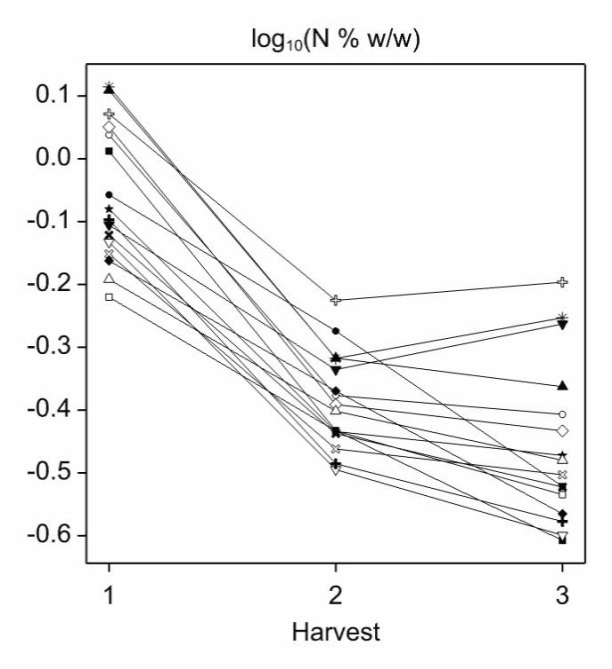


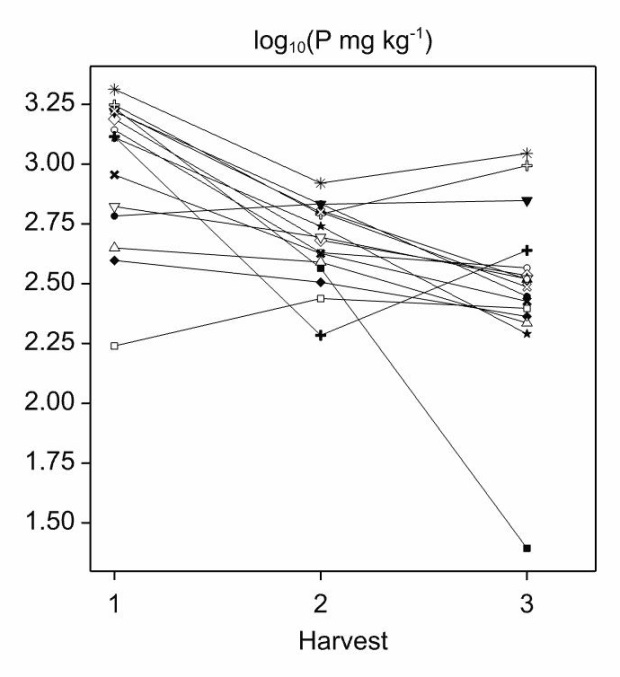

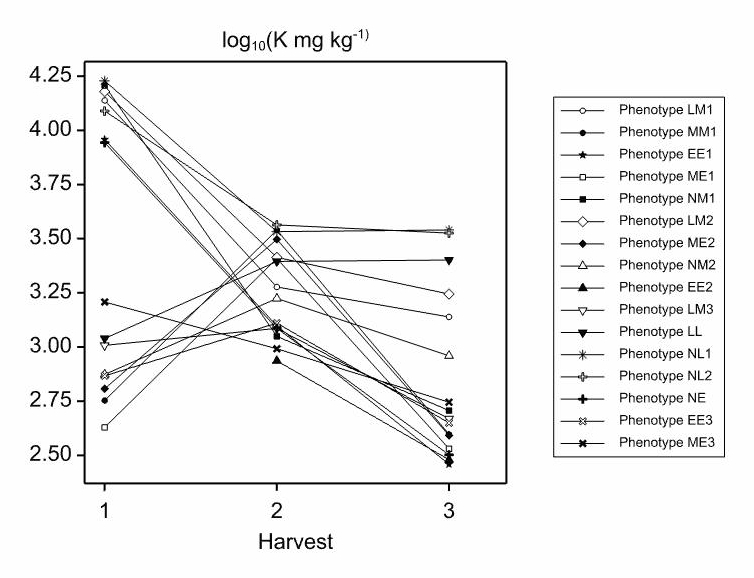


Supplementary Fig. 1. Genotypic trends and interactions with harvest for stem N, P, and K in diverse *Miscanthus* genotypes. Genotype categories are based on flowering and senescing phenotypes where the 1^st^ letter denotes flowering category (exertion of first flag leaf on or before: 21^st^ July 2009=early (E); 25^th^ August = mid (M)); after 25^th^ August = late (L), or not at all = non (N); 2^nd^ letter denotes senescence category based upon the loss of >80% greenness before: 23^rd^ October (E); 24^th^ November (M); or later (L).

Supplementary Fig. 2. Senescence progression (recorded as loss of greenness, where 10=100%) in early senescing genotypes, and genotype NM1, of *Miscanthus* grown in 2009 trait trial in Aberystwyth (Wales, UK). Central line indicates day of autumn harvest (23^rd^ October, 2009).
